# Thinning altered the optimum photosynthetic environment in a subtropical coniferous plantation

**DOI:** 10.1038/s41598-026-35052-0

**Published:** 2026-01-08

**Authors:** Shengtong Li, Mingjie Xu, Fengting Yang, Jiaxin Song, Xinyi Shi, Ziyi Wang, Huimin Wang, Xianjin Zhu, Chuanpeng Cheng, Jianlei Wang, Tao Zhang

**Affiliations:** 1 College of Agronomy, Shenyang Agricultural University, Shenyang, 110866 China; 2 National Critical Zone Observatory of Red Soil Hilly Region in Qianyanzhou, Key Laboratory of Ecosystem Network Observation and Modeling, Institute of Geographic Sciences and Natural Resources Research, Chinese Academy of Sciences, Beijing, 100101 China; 3 University of Chinese Academy of Sciences, Beijing, 100049 China; 4 College of Resources and Environment, Henan University of Economics and Law, Zhengzhou, 450046 China; 5 China Nonferrous Metals Industry Technology Development and Exchange Center Co. Ltd, Beijing, 100814 China

**Keywords:** Thinning, Forest, GPP, Optimum environment, Eddy covariance, Ecology, Ecology, Environmental sciences

## Abstract

Thinning offers long-term benefits for planted forests and is an important silvicultural practice. Meanwhile, ecosystem would respond to thinning and the optimal photosynthetic environment would change. Clarifying and quantifying the changing optimal photosynthetic environment induced by thinning is important for accurate prediction of carbon budgets and effective management of artificial forests. Based on six-year continuous in situ observations of carbon fluxes and corresponding environmental factors before and after 25% thinning in a typical subtropical plantation in China, this study revealed that thinning increased the optimum values of key environmental factors (net radiation (Rn), air temperature (Ta), and vapor pressure deficit (VPD)) for gross primary productivity (GPP). Meanwhile, thinning enhanced the ecosystem maximum GPP (GPP_max_) corresponding to each single optimum environmental factor. Among them, the highest GPP_max_, reaching 0.91 mg CO_2_ m^− 2^ s^− 1^, was found when Rn reached the optimum value and was 13% higher than that before thinning. In addition, the optimum photosynthetic environment configurations (the combination of multiple environmental factors) that could occur in the real world were detected and quantified. Before thinning, the optimal environment configuration was Rn = 692.85 J m^− 2^ s^− 1^, Ta = 23.43 °C, VPD = 0.96 kPa and SWC = 0.20 m^3^ m^− 3^, with a GPP_max_ of 0.98 mg CO_2_ m^− 2^ s^− 1^. After thinning, the GPP_max_ increased to 1.11 mg CO_2_ m^− 2^ s^− 1^, and the corresponding optimal photosynthetic environment configuration was Rn = 693.92 J m^− 2^ s^− 1^, Ta = 26.70 °C, VPD = 1.10 kPa and SWC = 0.21 m^3^ m^− 3^. All the values increased after thinning compared with those before thinning, and the changes in the optimal photosynthetic environment might be an important reason for the enhanced photosynthetic capacity of the thinned forests. These results indicated the positive responses of forest to proper thinning and could provide references for the development of forest management policies.

## Introduction

 Forests, as the most important terrestrial carbon sinks, have received increasing attention because of their role in mitigating climate change, and artificial forests are being planted worldwide^1^. Thinning is an important silvicultural measure for planted forests. Accompanying climate change, air temperature (Ta) is increasing^2,3^, and precipitation patterns are changing and are predicted to induce more frequent droughts^4–6^. Changes in environmental factors together with anthropologic management or disturbances would greatly affect the photosynthetic capacity of forests^7–9^. Therefore, it is important to clarify the optimum photosynthetic environment for forests, which could be important indicators for predicting and estimating the carbon sink function of forests.

The response of forest photosynthesis to environmental factors is expected to follow the general response pattern of individual plants, with an “increase-peak-decrease” pattern. Insufficient light probably constrains photosynthesis^10^, whereas excessive radiation can lead to photoinhibition^11^. Low temperatures may reduce enzyme activity, whereas high temperatures can cause enzyme deactivation^12^. Water deficiency would lead to stomatal closure^13^, and excessive water would result in root hypoxia^14^. In essence, for each environmental factor, there may exist an optimal value that maximizes ecosystem gross primary productivity (GPP). When an environmental factor deviates from its favorable range, either too high or too low, it will suppress GPP.

Previous studies have investigated the optimal Ta and optimal vapor pressure deficit (VPD) for forest photosynthesis at the leaf, canopy, and ecosystem scales^15–17^. However, these studies focused on the responses of ecosystems to a given environmental factor^18^. In reality, the photosynthetic response of forests is the result of the synergistic effects of multiple environmental factors, including light, temperature, and water availability^19^. When one environmental factor reaches its theoretical optimum, constraints imposed by other factors may lead to a significant reduction in actual GPP relative to expected levels^20^. Consequently, the optimum environment configuration (the combination of multiple factors) may not equal the theoretical optimum value detected for each single factor. The deviation of the optimal photosynthetic environment configuration from the optima of each single factor may arise from the interdependencies among environmental factors^13,21^, which should be considered when studies are conducted in real-world ecosystems. For example, subtropical China is under the control of a subtropical high-pressure system, which results in strong radiation, high Ta, a shortage of precipitation (PPT) and thus high VPD in summer^22^. These climatic conditions can markedly reduce plant stomatal conductance and substantially impair photosynthetic efficiency^23^. This implies that even when Ta reaches its optimal value in summer, the elevated VPD may still constrain vegetation photosynthesis, thereby preventing GPP from achieving its theoretical maximum. Consequently, relying solely on the optimal value of a single environmental factor is insufficient for accurately capturing the actual photosynthetic potential of forest ecosystems. It is essential to comprehensively evaluate the combined effects of multiple environmental factors, i.e., the optimal photosynthetic environment configuration^24^.

To enhance terrestrial carbon sinks and safeguard the ecological environment, extensive planted forests have been established across China since the 1980s^25^. However, most artificial forests are pure forests. After decades of growth, many problems have emerged^26,27^. Especially, owing to the single tree species and lower biodiversity, the resistance of artificial forests to natural disasters, pests and diseases is usually weak, accompanied by greater ecological risks^28,29^. Thinning is considered an inevitable approach to reconstruct artificial forests, which have long-term ecological and economic benefits^30,31^. Therefore, it is important to clarify the effects of thinning on the optimum photosynthetic environment; thus, researchers could accurately predict the changing carbon sink function of artificial forests.

Thinning could greatly affect the microclimate in forests^32,33^. After thinning, the leaf area index (LAI) decreases, and the removal of trees creates canopy gaps. Hence, light transmittance increases, which subsequently leads to the redistribution of light and heat resources in both the canopy and understory layers^34–36^. Simultaneously, reduced canopy interception enables more precipitation to reach the ground, thereby effectively alleviating water competition among plant^37^. Furthermore, thinning influences other environmental factors, such as wind speed, soil temperature, and soil water content (SWC)^38^. These changes in environmental factors likely promote adaptive adjustments in both trees and understory vegetation, enabling them to fully exploit the altered microclimatic conditions. Forrester et al.^39^ reported that thinning substantially increased the light use efficiency of individual trees, thereby improving the photosynthetic capacity of the remaining trees. Martin-Benito et al.^40^ reported that improved soil moisture conditions and increased stomatal conductance after thinning significantly increased the intrinsic water use efficiency of European black pine plantations, increasing their resilience to climate change. According to previous studies, thinning would probably alter the response characteristics of forests to environmental factors and their resource utilization strategies. The optimal range of these factors for forests inevitably shifts as habitat changes, affecting tree photosynthetic physiological processes and ultimately influencing ecosystem productivity^9,16^. The optimal photosynthetic environment involves complex interactions and mutual constraints among various environmental factors, thinning may alter the pre-existing synergistic relationships among these environmental factors and redefine the optimal environment for photosynthesis. Thus, the impact of thinning on the optimum photosynthetic environment may be more intricate and challenging to predict^13,21^.

Previous studies have focused on the impacts of thinning on environmental conditions or the functions of ecosystems^28,30^. However, few studies have explored the optimal environment configuration for GPP and clarified the effects of thinning on the optimum environment. Therefore, we conducted this study in a typical subtropical coniferous plantation in southern China, a region characterized by extensive planted forests and great contributions to the terrestrial carbon sink. In detail, this study aimed to (1) quantify the theoretical optimum values of each key environmental factor for pre- and post-thinning, such as net radiation (Rn), air temperature (Ta), and water conditions (atmospheric water conditions represented by the vapor pressure deficit (VPD) and soil water conditions represented by the soil water content (SWC)) at the ecosystem scale; (2) identify the optimum photosynthetic environment configuration (the synergistic combination of Rn, Ta, and VPD) that maximizes GPP under realistic conditions for pre- and post-thinning; and (3) determine the quantitative changes in this optimum environment configuration and the corresponding changes in GPP_max_ induced by thinning. This study would help elucidate the underlying mechanisms of the effects of thinning on forest photosynthetic capacity, thereby providing a theoretical foundation and practical guidance for the management of subtropical artificial forests and the enhancement of their carbon sink function.

## Materials and methods

### Site description

This study was conducted at the National Qianyanzhou Critical Zone Observatory of the Red Soil Hilly Region (QYZ for short) (26°44′29″N, 115°03′29″E, 102 m above sea level), which is located in a typical red soil hilly region in Jiangxi Province, China. It serves as a standard observation site within the China Flux Observation and Research Network (ChinaFLUX), featuring two forest flux towers-one established in 2002 and the other in 2008. QYZ is under the control of a typical subtropical monsoon climate. According to meteorological observations at QYZ from 1985 to 2014, the mean annual temperature is 18.0 ± 0.4 °C, and the mean annual precipitation is 1464.5 ± 308.8 mm. There are four seasons: spring (March‒May), summer (June‒August), autumn (September‒November) and winter (December‒February).

The vegetation at this site is a typical subtropical coniferous plantation. Before 1983, this region experienced severe soil and water erosion. To address this situation, trees were planted in approximately 1985, with pioneer and fast-growing species of Masson pine (*Pinus massoniana* Lamb.), Slash pine (*Pinus elliottii* Englem.) and Chinese fir (*Cunninghamia lanceolata* Hook.)^41^. The ecological environment has recovered and improved since then and has been a model of ecological restoration. However, after years of development, pure forests face many ecological risks, and proper forest management measures are needed to address this issue.

### Thinning treatment

The coniferous trees at the QYZ station were planted with a high density of 1460 stems ha^– 1^ in 1985. Therefore, many trees were suppressed and relatively small before thinning was conducted. To reconstruct and further improve the forest, the QYZ station conducted relatively large-scale thinning (~ 40 ha) in the winter of 2012 around the second flux tower (26°45′03.01″N, 115°03′36.24″E) established in 2008. For study purposes, pre-thinning observations were conducted from 2009 to 2012, which could be used as the reference for studying the effects of thinning. Given that forests change very quickly after thinning, to study thinning effects, observations from 2013 to 2014 were studied as post-thinning. In the thinned regions around the flux tower, Masson pine was the overwhelmingly dominant species, accounting for more than 85% of all the trees^42^. Other species, including Slash pine, Chinese fir and naturally growing broadleaf trees such as Schima superba, Liquidambar formosana, and Quercus acutissima, together account for less than 15% of the forests.

When thinning was conducted, approximately 25% of the basal area was removed. This intensity represents a moderately intensive thinning approach widely adopted in the management of subtropical planted forests in China. The commercially valuable parts (timbers) of the tree trunks were removed from the site, and most of the slash was left on the site. Tree stumps were also retained on site, with heights typically ranging from 25 to 35 cm. To study the effects of thinning on forests, some plots were also set around the tower. According to the plot investigation, before thinning, the mean stand density was 1329 stems ha^− 1^, and after thinning, it was 849 stems ha^− 1^ (Table 1). The stand structure characteristics, such as diameter at breast height (DBH), height of trees, and leaf area index (LAI), were also investigated and measured before and after thinning and are listed in Table 1. The LAI was calculated from the TRAC measurements by TRAC-WIN. Canopy openness of the canopy was analyzed from the digital hemispherical photographs by a Sidelook and Gap Light Analyzer (GLA).

**Table 1:** Stand structure characteristics for pre- and post-thinning. The values are the means ± SDs.

	Stand density (stems ha^− 1^)	Canopy openness (%)	Leaf area index (LAI)	DBH (cm)	Height (m)	Volume (m^3^ ha^− 1^)
Pre-thinning	1329	38.51 ± 2.16	2.94 ± 0.22	15.76 ± 5.39	13.40 ± 2.46	18.70 ± 2.16
Post-thinning	849	44.96 ± 1.36	2.27 ± 0.08	17.18 ± 5.53	13.94 ± 2.42	15.09 ± 2.21

### Observation and instrumentation

To investigate the effects of thinning, an eddy covariance (EC) flux observation system was installed at the QYZ site in 2008. After four years of observations, the thinning treatment was conducted in the region encompassing the footprint area of the flux observation. The EC measurement system was installed on the tower at a height of 18.0 m. The EC system included a 3D sonic anemometer (Model CSAT3, Campbell Scientific Inc., Logan, UT, USA) and an open-path CO_2_/H_2_O analyzer (Model LI-7500, LI-COR Inc., Lincoln, NE, USA). All signals were collected at a frequency of 10 Hz, and the CO_2_ and H_2_O fluxes were calculated and recorded at 30-min intervals by a CR3000 datalogger (Campbell Scientific Inc.).

The environmental factors were observed synchronously. Air temperature (Ta), water vapor pressure, and relative humidity were measured via a sensor (Model HMP45C, Vaisala Inc., Helsinki, Finland) in a ventilated solar radiation shield. The soil temperature and soil water content (SWC) were measured at a depth of 5 cm with a temperature sensor (Model 105 T, Campbell Scientific Inc., Logan, USA) and TDR probes (Model CS615-L, Campbell Scientific Inc.), respectively. Radiation was measured via a four-component net radiometer (Model CNR-1, Kipp & Zonen, Delft, ZuidHolland, Netherlands) and a pyranometer (Model CM11, Kipp & Zonen), and the net radiation (Rn) was calculated and recorded by the datalogger. Precipitation (PPT) was monitored with a rain gauge (Model 52203, RM Young Inc., Traverse, MI, USA). The environmental variables were sampled at 1 Hz, and 30-min average data were calculated and recorded by a CR1000 datalogger (Campbell Scientific, Inc.).

### Flux data processing

To ensure the reliability and consistency of flux data across China, the ChinaFLUX union has developed a series of standard methods for processing data^43^. Accordingly, in this study, the flux data were processed according to ChinaFLUX standard data processing methods^44^. The raw data were corrected by axis rotation to eliminate the possible influences of local terrain or instrument tilt. The Webb Pearman and Leuning correction (WPL correction)^45^ was used to eliminate disturbances introduced by water vapor and temperature. Spurious data caused by system failures or rainfall were removed when the value exceeded the given thresholds for different variables (e.g., for 30-min CO_2_ flux, the thresholds ranged from ‒3 mg CO_2_ m^− 2^ s^− 1^ to 3 mg CO_2_ m^− 2^ s^− 1^) and then when the anomalous data exceeded the mean ± 3SD. The storage fluxes were subsequently assessed and added to the flux values. To avoid possible underestimation of the fluxes under poor turbulent mixing conditions at night, nighttime (solar elevation angle < 0) data were excluded when the friction velocity (u*) was lower than the threshold calculated according to the method described by Reichstein et al.^46^. For the years 2009 to 2014, the average u* threshold was 0.18 m s^− 1^.

After the anomalous data were excluded, data gaps were generated. Data gaps were filled via the mean diurnal variation method and linear and nonlinear methods^47^. The observed daytime CO_2_ flux is the net ecosystem exchange (NEE), whose absolute value equals the net ecosystem productivity (NEP) but has the opposite sign (NEP = − NEE), and the nighttime CO_2_ flux equals the ecosystem respiration (Re), which can be extrapolated to the daytime using the Lloyd-Taylor equation to obtain the daytime Re. The GPP was subsequently calculated according to the equation GPP = NEP + Re. Further detailed information about the flux data correction and calculation can be found in previous relevant studies^48,49^.

### Data analysis

The carbon fluxes and corresponding climatic factors, including Rn, Ta, SWC and VPD were analyzed. To determine the optimum environment for ecosystem GPP, the data before thinning and after thinning were analyzed, respectively. The data were then binned according to each key environmental factor to detect the maximum GPP and the optimum environment. For example, to detect the optimum Rn for GPP, the data were binned into 18 groups, from low to high Rn, and the corresponding average GPP and Rn in each group were obtained. Then, the maximum GPP under the optimum Rn was detected. To determine the optimum environment configuration, the data were subsequently analyzed. For example, to detect the optimum Ta and VPD under certain Rn conditions, the data were first binned according to Rn and then according to Ta and VPD, respectively. The data corrections, calculations, statistical tests, and visualizations were conducted with MATLAB R2023a software (MathWorks Inc., Natick, MA, USA), PyCharm Professional 2023.1 (JetBrains s.r.o., Prague, Czech Republic) and Origin2021 software (Origin Lab Corporation, Northampton, MA, USA).

## Results and discussion

### The theoretical optimum values of key environmental factors before and after thinning

**Fig. 1:**
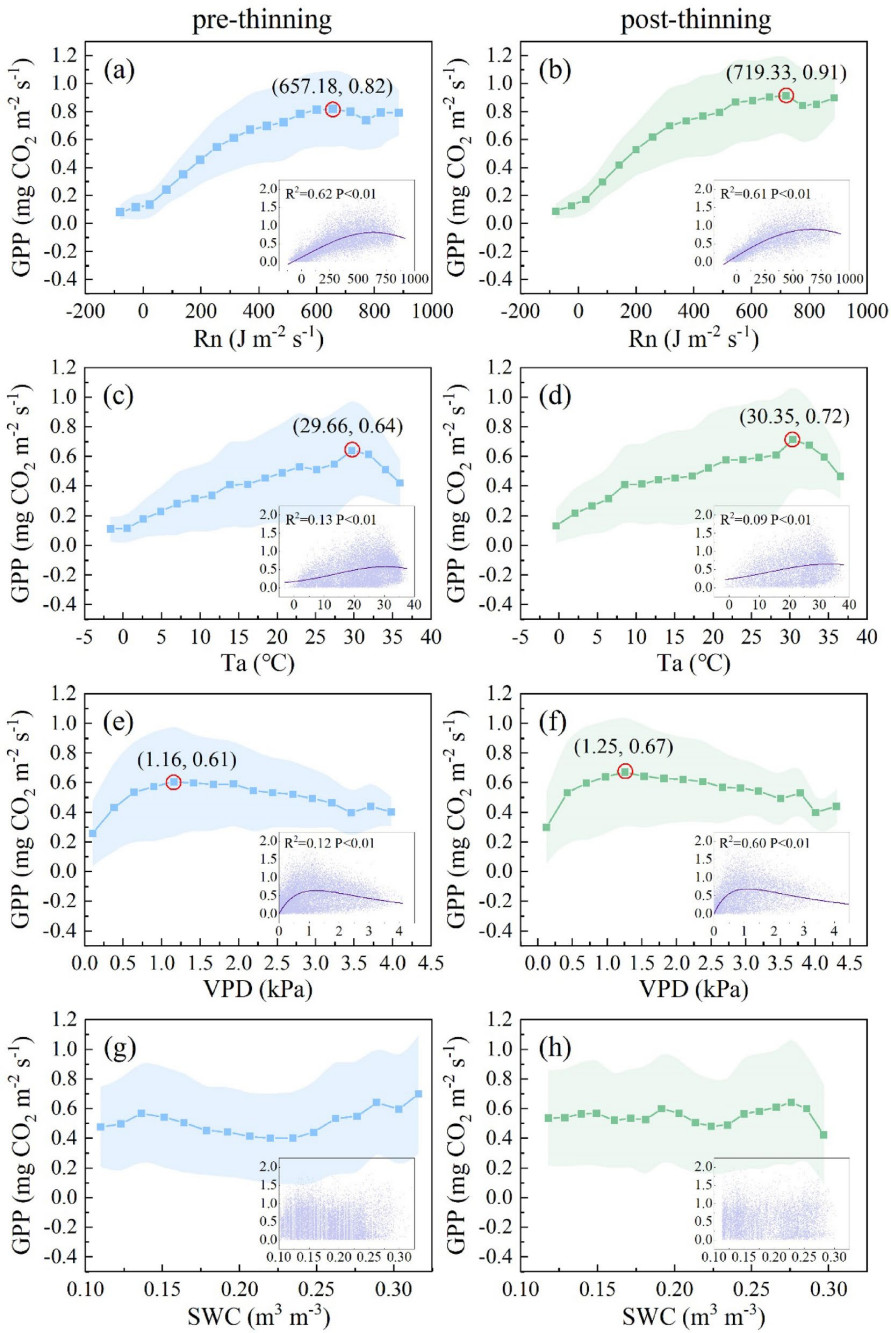
Responses of gross primary productivity (GPP) to environmental factors (net radiation (Rn), air temperature (Ta), vapor pressure deficit (VPD), and soil water content (SWC) at 5 cm depth) before and after thinning. The shadow regions represent the standard deviation. The red circles mark the maximum value points, and (x, y) indicate the optimal values of the environmental variables (optimal Rn (Rn_opt_), optimal Ta (Ta_opt_), and optimal VPD (VPD_opt_)) and the corresponding maximum photosynthetic capacity (GPP_max_).

Light, temperature, and water are essential natural resources for photosynthesis. A favorable environment would promote photosynthesis and increase GPP^50–52^. Theoretically, GPP would respond to each environmental factor with an “increase-peak-decrease” pattern^53^. That is, there would be a theoretical optimum value of each environmental factor for GPP. Thus, the responses of the GPP of this subtropical forest to each environmental factor, including Rn, Ta, VPD and SWC, were analyzed before and after thinning (Fig. 1). The results indicated that, except for SWC, the responses of GPP to Rn, Ta, and VPD followed the “increase-peak-decrease” pattern, as expected, which indicated the existence of an optimum value for GPP. Among them, the GPP was highest when Rn reached the optimum value (Fig. 1a and b), indicating the importance of Rn in this subtropical forest^54,55^. In addition, this result indicates this subtropical forest has carbon sink potential to be further explored, as Rn dominated the GPP without other environmental limitations.

The optimum value of each environmental factor for GPP increased after thinning (Fig. 1a–f). The optimum Rn (Rn_opt_) increased from 657.18 J m^− 2^ s^− 1^ to 719.33 J m^− 2^ s^− 1^, the optimum Ta (Ta_opt_) increased from 29.66 °C to 30.35 °C, and the optimum VPD (VPD_opt_) increased from 1.16 kPa to 1.25 kPa. These changes could be attributed to the redistribution of environmental resources and the changing vertical pattern^56^. According to previous studies, light transmittance increased after thinning, and understory light conditions improved because of the openness of the canopy^34^. Consequently, a decrease in canopy closure and improved ventilation may lead to thinned forests being light- and temperature-saturated under conditions of higher Rn and Ta^38^. Similarly, higher optimum Rn and Ta values were detected in the thinned forest in this study. In addition, the removal of trees would decrease competition for soil water, thereby improving soil water availability, as supported by the study of Cheng et al.^42^, which reported increased SWC following thinning at the QYZ station. The better soil water conditions may help mitigate the stomatal closure tendency induced by high VPD, allowing the forest to maintain higher photosynthetic rates under high VPD. This was evidenced by previous studies, which indicated that a properly high VPD tends to act as a pulling force for vegetation and has two edge effects. If the amount of soil water is sufficient, plants tend to open their stomata under proper high VPD and promote GPP. Otherwise, if there is a shortage of soil water, plants tend to close their stomata to prevent excessive water loss and the occurrence of physiological drought^57–59^. Therefore, owing to the likely increase in soil water availability, the higher VPD_opt_ after thinning found in this study is theoretically reasonable. We quantified the changes in the optimum values, which could be useful indicators for future forest carbon sink estimation.

Previous studies also indicated that thinning could alleviate the inhibitory effects of high temperature and low humidity on the stomatal conductance of the upper layer of a forest and thus promote photosynthesis in the remaining trees^60,61^. In addition, thinning increased the contributions of middle- and lower-layer leaves as well as understory shrubs to GPP^62^. These changes, accompanied by a decrease in competition for natural resources, may promote the maximum GPP (GPP_max_) under optimum environmental conditions^39,40,63^. After thinning, the GPP_max_ were found increased in this study, and the GPP_max_ under Rn_opt_ increased from 0.82 mg CO_2_ m^− 2^ s^− 1^ to 0.91 mg CO_2_ m^− 2^ s^− 1^, the GPP_max_ under Ta_opt_ increased from 0.64 mg CO_2_ m^− 2^ s^− 1^ to 0.72 mg CO_2_ m^− 2^ s^− 1^, and the GPP_max_ under VPD_opt_ increased from 0.61 mg CO_2_ m^− 2^ s^− 1^ to 0.67 mg CO_2_ m^− 2^ s^− 1^.

**Table 2:** Average values of each environmental factor (net radiation (Rn), air temperature (Ta), vapor pressure deficit (VPD), soil water content (SWC) at 5 cm depth) and maximum photosynthetic capacity (GPP_max_) under the optimum value of the given environmental factor (Rn_opt_, Ta_opt_, and VPD_opt_), which are marked in bold for both pre- and post-thinning.

Thinning treatment	Item	Rn (J m^− 2^ s^− 1^)	Ta (°C)	VPD (kPa)	SWC (m^3^ m^− 3^)	GPP_max_ (mg CO_2_ m^− 2^ s^− 1^)
pre-thinning	Rn_opt_	**657.18 ± 17.01**	28.85 ± 5.13	1.82 ± 0.71	0.17 ± 0.04	0.82 ± 0.27
	Ta_opt_	399.53 ± 230.61	**29.66 ± 0.65**	1.52 ± 0.50	0.17 ± 0.05	0.64 ± 0.33
	VPD_opt_	334.10 ± 235.60	25.10 ± 4.16	**1.16 ± 0.07**	0.17 ± 0.04	0.61 ± 0.37
post-thinning	Rn_opt_	**719.33 ± 17.53**	30.67 ± 3.58	1.95 ± 0.66	0.19 ± 0.05	0.91 ± 0.27
	Ta_opt_	427.29 ± 230.68	**30.35 ± 0.64**	1.55 ± 0.48	0.19 ± 0.05	0.72 ± 0.35
	VPD_opt_	356.22 ± 234.28	25.53 ± 5.02	**1.25 ± 0.08**	0.18 ± 0.05	0.67 ± 0.37

The GPP could be maximized, and its theoretical photosynthesis potential could be realized under Rn_opt_, Ta_opt_ and VPD_opt_. However, it is practically impossible for all three factors to reach their individual optima simultaneously because of natural environmental constraints. Thus, we wanted to know the values of other factors when a given factor achieved the optimum value (Table 2). Therefore, we can comprehensively analyze the most suitable environment that can be achieved in the real world. For both pre- and post-thinning, Ta was closest to Ta_opt_ under Rn_opt_. However, the VPD was higher under Rn_opt_. Consequently, under Rn_opt_, the coeffects of favorable light and temperature conditions would promote GPP^64^, but high VPD may depress GPP, as trees tend to close their stomata to prevent damage from physiological water deficiency^65,66^. Under Ta_opt_, Rn was obviously lower than Rn_opt_, and VPD was higher than VPD_opt_. Thus, in this case, Rn could not satisfy the light requirements of the forest, VPD depressed GPP, and the corresponding GPP_max_ was lower than GPP_max_ under Rn_opt_. Under VPD_opt_, Rn and Ta were both lower than the optimum values, thus leading to the lowest GPP_max_ among the three cases (i.e., Rn_opt_, Ta_opt_, and VPD_opt_).

According to the results in Table 2, when a single environmental factor reached its theoretical optimum, the other factors often deviated substantially from their respective optimum. Consequently, the GPP observed under such single-factor optimum conditions failed to represent the maximum photosynthetic capacity of the ecosystem^20^. However, we found that the GPP_max_ increased with the increasing number of factors approaching their optimal ranges. In this subtropical forest, both before and after thinning, this occurred under Rn_opt_, when the GPP_max_ was higher than that under Ta_opt_ and VPD_opt_.

### The optimum Ta combining with Rn and the thinning effects

Since it is difficult for Rn, Ta, and VPD to reach their optimal values simultaneously under natural conditions, we explored the combination of environmental factors that can maximize GPP under actual environmental conditions, that is, the optimal photosynthetic environment. According to the R^2^ values between GPP and the environmental factors, the correlation between GPP and Rn was significantly higher than that between GPP and the other environmental factors (Fig. 1). To further clarify the optimal photosynthetic environment configuration, we analyzed the response of GPP to Ta and VPD under different Rn conditions. The GPP did not change obviously with increasing SWC, which may be attributed to the influence of SWC being primarily manifested under drought conditions as a limiting factor, rather than exerting a direct promoting effect on GPP^67,68^. The underlying mechanisms through which SWC influences forest ecosystems differ from those of other environmental factors, and its response pattern may not follow the “increase-peak-decrease” pattern^68^. Therefore, the effects of SWC are not further discussed in this study.

**Fig. 2:**
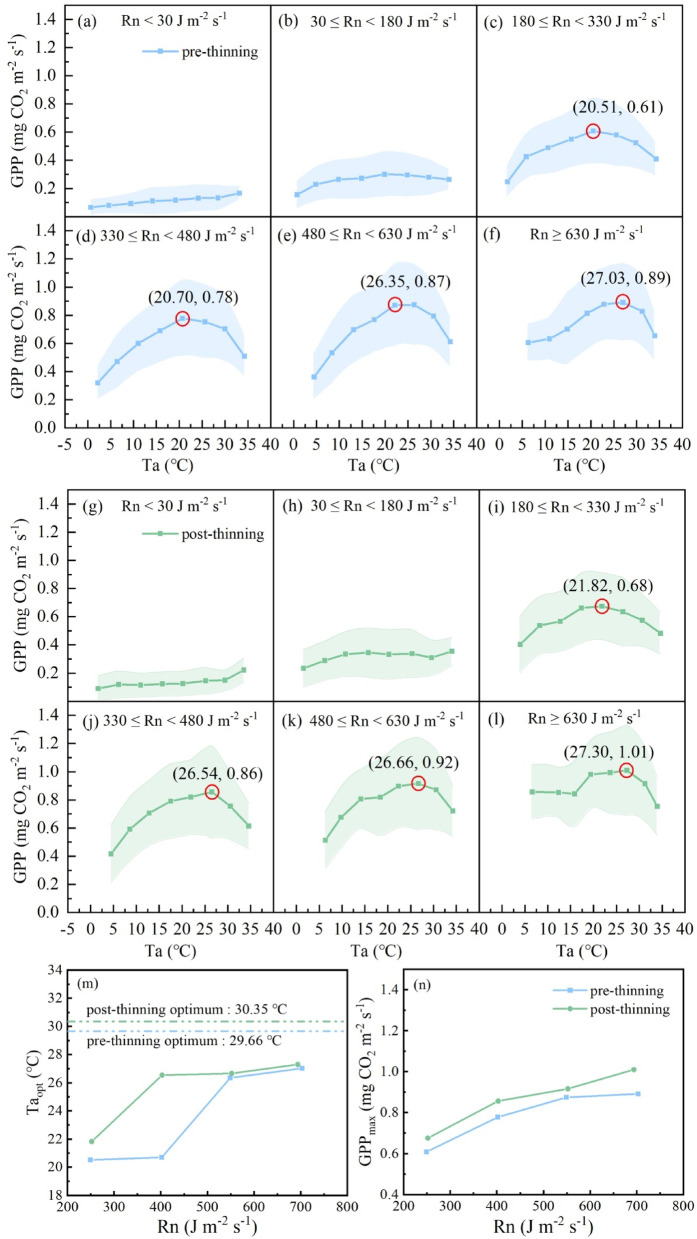
Responses of gross primary productivity (GPP) to air temperature (Ta) in different net radiation (Rn) classes before and after thinning. The shaded areas represent the standard deviation. The maximum values in each Rn class are marked with red circles, and the point (x, y) indicates the optimal Ta (Ta_opt_) and maximum photosynthetic capacity (GPP_max_) in different Rn classes. The trends of Ta_opt_ and GPP_max_ along with differing Rn classes are shown in panels (m) and (n).

The response of GPP to Ta has been widely reported, with a consensus on the existence of an optimum Ta under natural conditions^15,16^. However, when the analyses were conducted according to different Rn classes on the basis of the large dataset, interesting regulations emerged (Fig. 2). It could be found that when Rn was lower than 180 J m^− 2^ s^− 1^, the GPP did not exhibit an obvious response to elevated Ta due to insufficient light. This is reasonable, as the photosynthetic apparatus cannot capture sufficient photons; thus, the energy cannot sustain stronger photosynthesis^10,69^. When Rn was higher than 180 J m^− 2^ s^− 1^, the effects of Ta began to emerge. Ta_opt_ increased with increasing Rn both before and after thinning and approached the optimum values detected in Fig. 1 (Fig. 2m). Before thinning, Ta_opt_ sharply increased in the Rn class of 480 ≤ Rn < 630 J m^− 2^ s^− 1^, from 20.70 °C in the previous Rn class (330 ≤ Rn < 480 J m^− 2^ s^− 1^) to 26.35 °C (Fig. 2d and e). While after thinning, Ta_opt_ sharply increased to 26.54 °C in the Rn class of 330 ≤ Rn < 480 J m^− 2^ s^− 1^ (Fig. 2j), which occurred earlier than pre-thinning. The response divergence could be more directly observed in Fig. 2m, indicating that thinning enabled the forest to maintain higher GPP even under lower Rn conditions. This indicated an improvement in light use efficiency following thinning, as the vegetation exhibited enhanced responses to Ta without being constrained by light limitation. This finding is consistent with the findings reported by previous studies, which reported that plants could use weak light more efficiently after thinning^70,71^. As Rn increased, it approached the respective optima identified in Fig. 1c and d, which were 29.66 °C and 30.35 °C before and after thinning, respectively. Thus, the GPP_max_ increased in the higher Rn classes, and the values were always higher for post-thinning than pre-thinning (Fig. 2n), indicating that thinning enhanced the photosynthetic capacity of this coniferous forest.

In the step by step analysis progress for detecting the optimum photosynthetic environment, we could find that in the Rn class of Rn > 630 J m^− 2^ s^− 1^, when Ta reached Ta_opt_, the corresponding GPP_max_ (Fig. 2f and l) exceeded the values observed under Rn_opt_ alone (Fig. 1a and b). In detail, for pre-thinning, when Ta achieved Ta_opt_ of 27.03 ℃ under the circumstance of Rn > 630 J m^− 2^ s^− 1^, the GPP_max_ was 0.89 mg CO_2_ m^− 2^ s^− 1^ (Fig. 2f), which was higher than the GPP_max_ of 0.82 mg CO_2_ m^− 2^ s^− 1^ found under Rn_opt_ (Fig. 1a). For post-thinning, the corresponding Ta_opt_ was 27.30 ℃, and the GPP_max_ was 1.01 mg CO_2_ m^− 2^ s^− 1^ (Fig. 2l), which was higher than the GPP_max_ of 0.91 mg CO_2_ m^− 2^ s^− 1^ under Rn_opt_ (Fig. 1b). This finding indicates that identifying the “optimal photosynthetic environment configuration” (a combination of multiple factors that cooccur under natural conditions and maximize GPP) is more practically significant than focusing solely on theoretical single-factor optima. Notably, after thinning, the GPP_max_ under this “high radiation and optimal temperature” configuration was substantially higher than that before thinning. These findings indicate that thinning, by altering the canopy and vertical structure of the forest and affecting the microclimate, likely enhances natural resource utilization efficiency, thereby further improving the photosynthetic potential of the forest under optimal environmental configurations. We first confirmed the predominant role of Rn in determining the optimum photosynthetic environment, as shown in Fig. 1. To date, we have found a better environment configuration for GPP based on the single environmental optimum shown in Fig. 1. Furthermore, the combination of VPD and Rn for better environment configuration was studied.

### The optimum VPD combining with Rn and the thinning effects

**Fig. 3:**
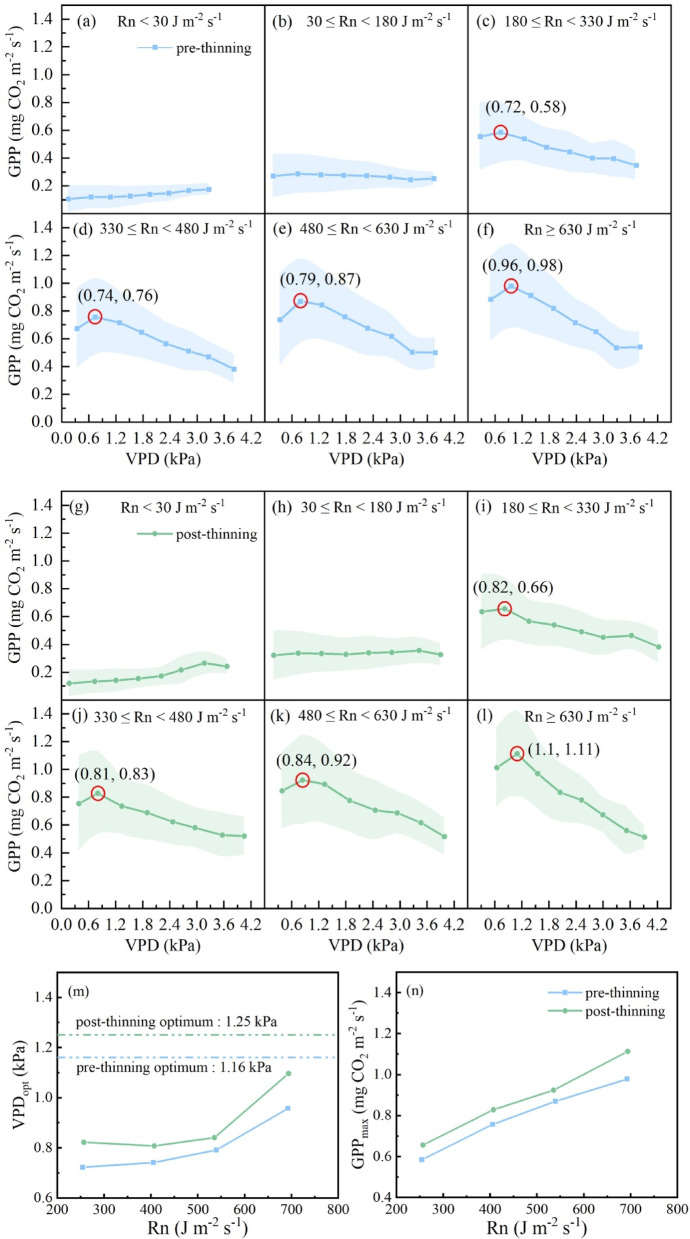
Responses of gross primary productivity (GPP) to vapor pressure deficit (VPD) in different net radiation (Rn) classes before and after thinning. The shaded areas represent the standard deviation. The maximum values in each Rn class are marked with red circles, and the point (x, y) indicates the optimal VPD (VPD_opt_) and maximum photosynthetic capacity (GPP_max_) in different Rn classes. The trends of VPD_opt_ and GPP_max_ along with differing Rn classes are shown in panels (m) and (n).

The responses of GPP to VPD under different Rn conditions were analyzed both before and after thinning (Fig. 3). When Rn was lower than 180 J m^− 2^ s^− 1^, the GPP remained low and did not change with increasing VPD, indicating the dominant control effects of Rn on ecosystem photosynthesis^54^. In the middle and high Rn classes, the influence of VPD on GPP became evident. The GPP increased with VPD until VPD_opt_ and then decreased, following the expected pattern, as previously indicated by the existence of a response peak^65^. As Rn increased, VPD_opt_ increased both before and after thinning, approaching the theoretical single-factor optimum as previously identified (Fig. 3m). Consequently, the GPP_max_ increased in the higher Rn classes (Fig. 3n). It could be found that Ta_opt_ and VPD_opt_ simultaneously got to their respective optimal values in the higher Rn classes, which supported the predominant role of Rn in regulating GPP (Figs. 2m and 3m).

The post-thinning VPD_opt_ and corresponding GPP_max_ values were always higher than those before thinning across all Rn classes (Fig. 3n). This finding indicates that, to some extent, thinning alleviated the adverse effects of high VPD-induced atmospheric drought stress^23,72^. In combination with reduced soil water competition due to tree removal^61,73^, a moderately high VPD likely contributed to higher GPP. This is reasonable, as relatively high VPD under sufficient soil water availability promotes stomatal opening; therefore, more CO_2_ enters the photosynthetic apparatus and improves light and heat use efficiency, ultimately increasing GPP^57,74,75^.

For the optimum environment configuration, before thinning, we found that in the class of Rn > 630 J m^− 2^ s^− 1^, when the VPD_opt_ was 0.96 kPa, the corresponding GPP_max_ could reach 0.98 mg CO_2_ m^− 2^ s^− 1^ (Fig. 3f), which was higher than the GPP_max_ found under sole Rn_opt_ and the previously mentioned Ta_opt_ under Rn > 630 J m^− 2^ s^− 1^ (Figs. 1a and 2f). Thus, this case might refer to the circumstances under which the optimum environment configuration occurred. Similarly, for post-thinning, the corresponding VPD_opt_ was 1.10 kPa, and the GPP_max_ was found to have the highest value of 1.11 mg CO_2_ m^− 2^ s^− 1^ (Fig. 3l), which was higher than that under sole Rn_opt_ and the previously mentioned Ta_opt_ under Rn > 630 J m^− 2^ s^− 1^ reported in Figs. 1b and 2l.

### The optimal photosynthetic environment and the changes induced by thinning

**Table 3:** Comparisons of the optimal photosynthetic environment (net radiation (Rn), air temperature (Ta), vapor pressure deficit (VPD), soil water content (SWC) at 5 cm depth) and maximum photosynthetic capacity (GPP_max_) before and after thinning.

Thinning treatment	Rn (J m^− 2^ s^− 1^)	Ta (°C)	VPD (kPa)	SWC (m^3^ m^− 3^)	GPP_max_ (mg CO_2_ m^− 2^ s^− 1^)
pre-thinning	692.85 ± 51.38	23.43 ± 5.71	0.96 ± 0.14	0.20 ± 0.04	0.98 ± 0.30
post-thinning	693.92 ± 53.96	26.70 ± 4.16	1.10 ± 0.13	0.21 ± 0.05	1.11 ± 0.31

The optimum photosynthetic environment for pre-thinning and post-thinning could be identified according to the previous analyses when GPP_max_ reached the maximum value. The averages of other factors were subsequently calculated to provide accurate quantified indicators for this subtropical forest. According to the results in Table 3, the optimum photosynthetic environment that can be achieved in the real world for pre-thinning was Rn = 692.85 J m^− 2^ s^− 1^, Ta = 23.43 °C, VPD = 0.96 kPa and SWC = 0.20 m^3^ m^− 3^, with a corresponding GPP_max_ of 0.98 mg CO_2_ m^− 2^ s^− 1^. After thinning, the optimum photosynthetic environment changed to Rn = 693.92 J m^− 2^ s^− 1^, Ta = 26.70 °C, VPD = 1.10 kPa and SWC = 0.21 m^3^ m^− 3^, with a corresponding GPP_max_ of 1.11 mg CO_2_ m^− 2^ s^− 1^.

According to the results, the optimum photosynthetic environment that can actually be achieved in reality does not completely align with the optimal values of each single environmental factor (Tables 2 and 3). Generally, the values of each environmental factor in the real optimal photosynthetic environment are generally lower than their respective single-factor optimal values, indicating that the optimal photosynthetic environment is by no means a simple combination of the optimal values of each environmental factor but rather a complex combination regulated by mutual influences^13,21^.

The values of each environmental factor in the optimum photosynthetic environment after thinning were all higher than those before thinning, indicating that thinning could improve the resource utilization capacity of the initially overly dense forest ecosystem^39,40,63,76^; thus, the GPP_max_ was greater after thinning (Fig. 3). This was consistent with the results of the single-factor optimal values, which proved that thinning improved the microclimate environment, enhanced the resource utilization capacity of vegetation, and thereby increased the photosynthetic capacity of the forest.

## Conclusions

The photosynthetic capacity of vegetation is greatly determined by environmental conditions, and the extent to which it can achieve maximum photosynthesis depends on how favorable those conditions are. However, under natural conditions, the specific optimal values of each environmental factor for GPP rarely occur simultaneously. Therefore, based on clarification of the optimal values of each single factor, this study further conducted a comprehensive analysis to identify the optimal photosynthetic environment configuration (including multiple environmental factors) that this subtropical planted forest ecosystem can achieve under natural conditions before and after thinning. Before thinning, the optimum photosynthetic environment configuration was Rn = 692.85 J m^− 2^ s^− 1^, Ta = 23.43 °C, VPD = 0.96 kPa and soil water content (SWC) = 0.20 m^3^ m^− 3^. After thinning, the optimum configuration changed to Rn = 693.92 J m^− 2^ s^− 1^, Ta = 26.70 °C, VPD = 1.10 kPa and SWC = 0.21 m^3^ m^− 3^. Correspondingly, the GPP_max_ increased from 0.98 to 1.11 mg CO_2_ m^− 2^ s^− 1^ after thinning. The optimum values of key environmental factors for ecosystem photosynthesis all elevated after thinning. This might be one of the reasons why thinning increases the carbon sequestration capacity of this subtropical plantation. In addition, for both pre- and post-thinning, the GPP_max_ under the optimal photosynthetic environment configuration was always higher than the GPP_max_ when a single factor reached its optimum. These results manifested that thinning elevated the optimum environmental conditions and ecosystem photosynthetic capacity, and the optimum environment configuration that realistically occurs in the natural world is a more important indicator of GPP_max_ compared to the single-factor optimum, which should be taken into more careful consideration when assessing the impact of climate change on the carbon sink function of terrestrial ecosystems.

## Data Availability

Data of this study are available from the corresponding authors upon reasonable request.
